# Global Health Initiatives and aid effectiveness: insights from a Ugandan case study

**DOI:** 10.1186/1744-8603-7-20

**Published:** 2011-07-04

**Authors:** Valeria Oliveira Cruz, Barbara McPake

**Affiliations:** 1 Department of Global Health and Development, London School of Hygiene & Tropical Medicine, Keppel Street, London, WC1E 7HT, UK; 2 Institute for International Health and Development, Queen Margaret University, Edinburgh, Musselburgh, EH21 6UU, UK

## Abstract

**Background:**

The emergence of Global Health Initiatives (GHIs) has been a major feature of the aid environment of the last decade. This paper seeks to examine in depth the behaviour of two prominent GHIs in the early stages of their operation in Uganda as well as the responses of the government.

**Methods:**

The study adopted a qualitative and case study approach to investigate the governance of aid transactions in Uganda. Data sources included documentary review, in-depth and semi-structured interviews and observation of meetings. Agency theory guided the conceptual framework of the study.

**Results:**

The Ugandan government had a stated preference for donor funding to be channelled through the general or sectoral budgets. Despite this preference, two large GHIs opted to allocate resources and deliver activities through projects with a disease-specific approach. The mixed motives of contributor country governments, recipient country governments and GHI executives produced incentive regimes in conflict between different aid mechanisms.

**Conclusion:**

Notwithstanding attempts to align and harmonize donor activities, the interests and motives of the various actors (GHIs and different parts of the government) undermine such efforts.

## Background

Over the past decade, the international aid community has shown greater concern with improving aid effectiveness. In spite of historical gains in health status, challenges still abounded: in 1998, the infant mortality rate (IMR) in Africa was still 91 per thousand, more than four times the rate for Europe [1]; in 2006, over 3.3 billion people worldwide were at risk of malaria transmission contributing to approximately 1 million deaths each year [2]; and the estimated number of individuals living with HIV/AIDS by 2001 in Sub-Saharan Africa was 28.5 million. The failure to effectively deliver available interventions largely accounts for the excess mortality among the poor [3]. The international aid community thus sought for new "ways of doing business" that could tackle the high burden of disease in the low-income world by expanding access to interventions such as vaccines, insecticide treated bed nets, and anti-retroviral therapy. A range of targets, agreements, and partnerships emerged. Among these were the Roll Back Malaria Partnership established in 1998, the Millennium Development Goals (MDGs) adopted in 2000, the Global Fund to Fight AIDS, Tuberculosis and Malaria (Global Fund) created in 2002, and the Paris Declaration on aid alignment and harmonization agreed in 2005.

Over this period the term Global Health Initiatives (GHIs) started to be used. Other terms that appear to label an overlapping set of phenomena are Global Public Private Partnerships [4,5] and Global Health Partnerships [6]. A general definition of GHIs is still subject to discussion [7]. A useful one for the purposes of this paper describes GHIs as a standard model for financing and implementing disease control programs in various countries and in different regions of the world; they can be part of a multilateral or a bilateral program as the case of PEPFAR (the United States President's Emergency Plan for AIDS Relief); alternatively they can be established as a public private partnership like the Global Fund [8]. It is estimated that more than 100 such entities exist [9].

GHIs have tended to support the involvement of non-state actors, initially the private or commercial sector and later also civil society organizations, thus bringing diversity in the range of stakeholders involved in the health sector. While the majority of these initiatives aim at galvanizing support-financial technical and political-to low-income countries, their remit differs as some focus on advocacy and others operate as funding bodies. An example of an advocacy initiative is the 'Countdown to 2015' working at the global level to track progress made towards the achievement of the MDGs 1 (Eradicate Extreme Poverty & Hunger); 4 (Reduce Child Mortality); and 5 (Improve Maternal Health); to promote the use of evidence in policy making; and to increase health investments at the country level [10]. An example of a GHI operating as a funding body is the Global Alliance for Vaccines and Immunization (GAVI). It provides financial and in-kind support to developing countries in order to increase access to vaccines and to support sustainability of national efforts to control childhood diseases responsible for high mortality [11].

The amount of financial resources provided by GHIs for scaling up specific health interventions in low and middle income countries has been unprecedented. Combined, the Global Fund and PEPFAR have disbursed over US$26 billion since their creation for HIV/AIDS prevention and treatment [12,13]. The United States President's Malaria Initiative (PMI) committed over US$1.25 billion between 2006 and 2010 to 15 countries in Sub-Saharan Africa [14]. These additional resources raised expectations. For example, plans for the eradication of diseases such as malaria and measles are now discussed by the international health community, but were not considered options a decade ago when funding for research, development and expanded access to effective interventions was scarce.

However, when resources are earmarked to fund specific health interventions as is the case with the way many GHIs operate, they may create problems at country level, mirroring those faced by the project approach: narrow targets [15], fragmentation and duplication of efforts; and pressure on governments to respond to the separate requirements of different programs and donors [16]. Overall, evidence about the operation of GHIs is still scarce. Some studies have focused on quantitative analyses of disease outcomes [17-22] while others have started to shed light on the immediate effects of GHIs on health systems [7,9,15,23]. However, the robustness of the latter studies has been constrained by the lack of use of theoretical frameworks which would have helped in providing more rigorous accounts as to the behaviours of the actors involved in the delivery of aid.

This paper presents the results of a study on two GHIs-PEPFAR and the Global Fund-in the early stages of their operation (2003/2004) in Uganda. These two GHIs became very prominent, significantly funding actors in the country. The paper seeks to provide an in-depth examination of the behaviour of actors associated with the two GHIs and the responses of the government of Uganda (and its various parts). It adopts agency theory as a conceptual framework to understand these behaviours and to explain the underlying incentive regime of the relationships between these actors.

### Methods and analytical framework

The results reported in this paper form part of a larger study that set out to better understand the relationship between donors-bilateral and multilateral agencies-(including GHIs) and the government of Uganda (and its various parts). The importance of real-life context is captured by qualitative research in general [24] and in particular by a case-study approach [25]. An in-depth qualitative and case study approach thus was required to investigate the complex subject of the governance of aid transactions in Uganda, given the small number of organizations (sample size) involved. The findings presented in this article represent the sub-set of the data collected concerned with GHIs. Data collection took place in Uganda from September 2003 to June 2004. This included:

a) A total of 36 in depth and semi-structured interviews were conducted with policy makers and officials from donor agencies based in Uganda at national level. The selection of interviewees was purposive [26] combined with snowball technique [27]. Out of the 36 interviewees, five were key informants. Key Informants provided expert knowledge about the relationship between the parties; they were accessed over the course of the project, and were more reflective than other respondents [27]. Interviews were conducted using both formal/semi-structured guides as well as informal, unstructured conversations.

b) Observation of 30 government/donor (including GHIs) meetings took place at national level. These included joint review missions, public expenditure reviews and project evaluations. They covered not only facts but also observations of interactions and behaviours. Although the work focused on the national level interactions between the government and donors, district and civil society views were partially captured through discussions as observed during meetings.

c) Various policy documents (e.g. memoranda of understanding between the parties and annual performance reports) were collected and analyzed. Both published and unpublished documents relevant to the research topic were collected.

The analytical process involved: familiarization with the data (including data cleaning and checking for consistency), development and application of a coding scheme (or indexing) based on the identification of a thematic framework, charting and interpretation [26-28]. Agency theory (see below) guided the conceptual framework of this study and was used to generate the first set of themes to code the data. Amendments were made according to themes revealed by the data.

Agency theory can be used to understand economic relationships. The basic model comprises two individuals: a principal and an agent. In this relationship, there is an explicit or implicit contract between the parties, and as in any contract, principals use incentives to guide or to motivate the agent's actions towards agreed desired outcomes. The principal will contract and compensate an agent for the costs or disutilities associated with the agent's implementation of an agreed activity, leading to the advance of the principal's objective function.

In the context of international development assistance, the value of such a framework lies in its ability to understand the incentive structure embedded in the aid delivery process [29]. But incentives can only be understood by reference to the motivations of actors. For example, a reassignment of responsibilities can only be understood as punishment or reward (or neither) in the light of understanding of the motivation of the person reassigned. This study sought to understand the motives of the relevant actors in order to identify and analyze the incentives present in implicit and explicit contracts. The interpretation of organizational objective functions relied on the observation of the behaviours of relevant actors throughout the field work and interviews. Two approaches could have been taken: one would be to discover the nature of the agency relationships; another is to use agency framework as a mode of analysis. In this paper we opted for the latter, and used agency theory as a theoretical framework to seek explanations for outcomes observed and the incentive regime that has been put in place; rather than testing the hypothesis of there being or not a principal agency relationship.

In order to ensure reliability [30,31], objective and comprehensive records of the data generation and analytical processes were maintained. Respondent validation [32] was sought by presenting the preliminary research findings during a dissemination workshop in Uganda in October 2005. Deviant case analysis [30,33] was incorporated into the analytical process of this research. The triangulation of different data sources (interviews, observation and documentary analysis) was carried out to allow for one source balancing the scope for errors and bias of the other [34]. Ethical clearance was obtained from the London School of Hygiene and Tropical Medicine, the Institute of Public Health/Makerere University and the National Council for Science and Technology in Uganda. Consent for interviews was agreed verbally. An information sheet was given to every interviewee. Confidentiality of data was maintained throughout the research process and no names of individuals interviewed were disclosed.

## Results

### Health development aid in Uganda

The government of Uganda stated its preference for donor funding to be channelled through the general or sectoral budgets (instead of project support) on the basis that these should be more efficient, equitable and should allow them greater ownership [35]. Introduced in Uganda in 1998, budget support occurred in two different forms: general contributions to the budget of the government and earmarked contributions to the Poverty Action Fund (PAF)-equivalent to a Poverty Reduction Strategy Paper (PRSP). The number of donors contributing to budget support increased from five in 2000/01 [36] to 12 in 2002/2003 [37]. Sectoral budget support to the health sector in Uganda was launched in 2000 in the form of a SWAp (Sector Wide Approach), under which donors and government pooled resources, and jointly agreed the National Health Policy (NHP) and the Health Sector Strategic Plan (HSSP) and exercised oversight over their implementation. The proportion of funding for the health sector financed through projects decreased from 45% in 1999/00 to 34% in 2002/03 in relation to the overall resource envelope for the health sector [38].

However, project funding started to increase once again from 2003 onwards as Uganda became a recipient of large volumes of funds from GHIs, mainly focused on HIV/AIDS. Over the period covered by this research (from 2003 to 2004), the total approved budget by the Global Fund to Uganda totalled US$160.6 million^1 ^[39]. PEPFAR's budget for Uganda in 2004 was US$94 million [40]. By February of that year, 40% of the PEPFAR budget had been disbursed [41], indicating fast disbursement. In comparison the government budget for the entire health sector in financial year 2004/2005 was US$136.5 million [42]. This amount included budget support contributions.

### Structural features of the two GHIs

PEPFAR funds could not be provided directly to the government, only to non-governmental and private sector organizations (legal requirements established through the US Congress). In contrast the Global Fund operated as a financial instrument based on proposals being led by the government of Uganda. The Global Fund mechanisms for fund disbursement are somewhat flexible and in countries like Mozambique it used a common basket of pooled funds contributed by various donors and managed by the government [43].

In Uganda, both PEPFAR and the Global Fund opted to create parallel systems of management. Neither of these were seen to have contributed to the health Sector Wide Approach (SWAp), a mechanism which would have earmarked their funds for the health sector, but otherwise left the financial control in the hands of the government, overseen by a collective of bilateral and multilateral agencies. The Global Fund in Uganda used a separate project management unit within the Ministry of Health (MoH), their own monitoring tools (rather than the common mechanisms adopted through the Joint Review Missions, a performance review mechanism under the SWAp) and a parallel system for procurement (although the Global Fund guidelines made provision for the use of a common working arrangement).

Therefore neither Global Fund nor PEPFAR participated in the common technical mechanism of aid coordination among health sector stakeholders. Their proposals were not scrutinized by the Sector Working Group, set up by the Ministry of Finance Planning and Economic Development (MoFPED) and MoH to assess projects for value for money and alignment with government policies and plans. PEPFAR also followed their own funding and audit timetable instead of the national schedules for planning and budgeting.

Another special requirement set up by the Global Fund was the conditionality of additionality: the funds it provided had to be additional to those budgeted nationally and should not be treated as fungible. However, this condition came into conflict with macroeconomic budget ceilings set by the MoFPED. If these ceilings had been reached, the offer of resources from the Global Fund should in principle have been rejected. While there were discussions to apply the ceiling to Global Fund Round four, these did not materialise in the end [44,45].

During interviews some respondents explained that the rationale that drove GHIs like PEPFAR and the Global Fund to set up these parallel mechanisms were related to the weak capacity of government-particularly in relation to timely disbursement of funds, procurement and monitoring and evaluation. If they had decided to work through the existing government structures, this would have delayed the implementation schedule of their activities. Interviewees also said that separate management structures were used as a mechanism to reduce fiduciary risks. The latter was substantiated to some extent when in 2005, the Global Fund identified serious mismanagement problems in five of its grants to Uganda leading to their suspension [46]. However the suspension was lifted later in the year highlighting some of the complexities related to this issue-further explored elsewhere [47].

### Behaviour of GHIs and incentives

PEPFAR was argued by a number of key informants and government officials to be detrimentally affecting the health system. Competition for human resources was a particular concern. Often mentioned was the loss of highly qualified staff to PEPFAR funded projects in the face of higher salaries and benefits. This problem was said not to be restricted to government units but also to affect the private not-for-profit sector (which receives financial subsidy and seconded health workers from the government).

Staff were said to be moving primarily to two specific organizations receiving support from PEPFAR. One of them received 300 applications for clinical positions advertised in early 2004. Salaries paid by this organization were reported to be three times those paid by the private not-for-profit sector. The view of an interviewee from one of these organizations was that:

*"We are not poaching staff; applicants are not from government units. But on the other hand, it's a free world"*. (Private sector representative)

It was reported that the targets set by the US government for PEPFAR were not chosen in consultation with local government partners. Furthermore, in contrast to the disclaimer in the Memorandum of Understanding between the government of Uganda and Health donors that "*as provided in the Constitution of Uganda*, [both parties should] *ensure that other marginalized groups of society such as the poor, the displaced and the disabled are specifically addressed" *[48], PEPFAR did not outline a clear strategy on how it would reach these particular groups. It did not explicitly mention a focus on the poor, only on orphans. A common critique made in various meetings of health sector stakeholders was that the agencies implementing PEPFAR projects were reaching their targets by focusing on 'easy to reach' population groups such as health workers, teachers, police officers in large urban areas as opposed to the poor and vulnerable in rural parts of the country.

### Conflicting motives of parties

The Global Fund system requires the recipient country to apply for funding. Uganda applied for, and was successful in securing funding in all four rounds within the period of fieldwork. The justification used by those leading the application process in the MoH was the under-funding of the sector. The volume of funding made available by the Global Fund, and its perceived accessibility seemed to make certain members of the government more flexible about its rules and mechanisms leading them to interfere with the existing integrated budgeting processes, it was argued.

*"People rushed off around the Global Fund but collectively the rest of us *[budget support donors] *have more money that we are providing to the budget *[49]^2^. *But that is not seen to be accessible in the same way ... somehow the idea of the Global Fund money even if it's relatively small, excited much more political interest. I don't know ...it's seen as an opportunity that anybody can get something out of it and somehow with budget support money that isn't *[the case]". (Donor representative)

Various donors reported that they had been willing to increase their contributions via general budget support to the government (and consequently to the health sector) but had been prevented from doing so by the MoFPED on the basis of the country's macroeconomic budget ceilings.

Hence it would appear that some members of the government went to considerable lengths to attract additional funding from a source not operating through the mechanism which the government had stated it preferred, while other members rejected funding from sources operating through that mechanism. An explanation of this apparently perverse behaviour was offered:

*"The Ministry of Finance encourages the use of SWAp, but currently the approach of the Ministry of Finance [with]... sectoral budget ceilings results in threats to the sector, not only because there is insufficient funding to the sector at the moment, but also because it encourages the sector to seek funds elsewhere, off budget. If the sector was getting sufficient or a lot more funds through the budget it would be easier to argue against the GF and other GHIs"*. (Technical assistant)

When disagreements occurred between the government and GHIs, for example because of the lack of alignment with sector plans, the government did not always operate as a single entity. The PEPFAR program was agreed directly with the President's office without much scope for inputs from health sector stakeholders.

*"If the president has said yes, then *[a senior health sector official] *saying hang on, not like that, isn't going to get us anywhere, he can't even be confident that his ministers are saying the same thing as him"*. (Donor representative)

The application to Global Fund round four was another indication of non-alignment of motives within government but also between government and different donors. The budget support and SWAp donors criticized government for applying to the Global Fund yet when donors were consulted during a monthly coordination meeting at the time of round four there had been general agreement in favour of the government applying (though no discussion took place at the time of the meeting as to how the funds should be channelled-via the SWAp or through a project management unit).

### Weak institutional environment

A number of institutional issues represented an added layer of complexity in the relationship between the GHIs and the government, most prominently with regard to changes in authority and leadership as well rules and regulation which came into conflict.

The problem of lack of alignment with the stated goals of government was said to be related to lack of authority within government combined with a perceived low level of commitment by senior management [which was seen to be detached from the routine management of the technical programmes, lacking knowledge of their activities and not showing ownership (key informants)] and a lack of strong institutions-which instead made the system rely on key individuals. The waning commitment to SWAp and general budget processes and objectives over the period of the fieldwork seems to exemplify these arguments.

Some members of government argued that the rules and regulations of the public bureau would be sufficient to align incentives in this environment. The perception was that because government was a bureaucracy, the policies and rules it had effected would be adhered to.

*"We have clearly said that our preferred mode of financing is budget support, and over the years, budget support has been on an increasing trend and even to provide more incentives for ministries, the issue of integrating projects into the budget is meant to be a trade off. If you have more projects then you have less budget support-as government we are not very much in control of projects so need to think twice if they are worth it....the way government works*, [is that] *it's a bureaucracy, so there, are no power struggles in that sense"*. (Government official)

However, it seemed that rules and regulations (e.g. the SWAp related structures like the Sector Working Group) put in place by government have not been able to curb project expansion and ensure that technical programs adhered to the budget system.

The SWAp appeared to be a major objective of the majority of development agencies during its introduction and first years of implementation in Uganda (between 2000 and 2002/03).

*"The range of individuals motivated by the SWAp made the realization of the SWAp benefits easier...which was strengthened by the continuity of committed individuals in key donor agencies and posts..." *(Donor representative)

This cohesion was reported to have subsided by late 2003 with the introduction of the large GHIs (PEPFAR and Global Fund) which were more strongly driven by disease-specific goals.

*"In 2001 the overall balance was towards integration and coordination and two years further down the road and the arrival of alternatives in the form of the Global Fund and other projects has actually deepened the conflict and thrown that balance off*." (Technical assistant)

Within the structure of the MoH in Uganda there were two directorates. The technical programs fell under the Directorate of Clinical and Community Services while Directorate of Planning and Development was in charge of coordination. In line with its role, the Directorate of Clinical and Community Services was said to follow a more vertical, disease-specific approach, often preferring the project mode (technical assistant). Consequently, there had been some level of tension which the leadership of the MoH was said to have managed to balance during some periods. The previous Minister (at the time of fieldwork) seemed to have played a major role in aligning staff motives within government through his vision, authority and charisma as a political leader.

*"He was really engaged with the sector and he would get everybody *[to] *line up in one direction"*. (Government official)

*"I have seen people listening to *[the previous minister's] *ideas about health sector reforms until 9 o'clock at night"*. (Donor representative)

However as there was a change of Minister, there was perceived to be a change in which directorate had more authority in the conflict, explaining changing support levels for SWAp vis-à-vis GHIs. Some key informants observed that at the outset the technical programs were the ones seeking or accepting project funding (including from the Global Fund), but after late 2003 it was said that senior management of the Ministry were more actively involved as well. One government official said: "*some of the new people seem not to value the *[SWAp] *partnership to the same extent"*. Those who remained supportive of the SWAp principles and structures were said to be few by the time of field work.

To a certain degree, the observed shift away from the SWAp and towards projects was related to new leaders taking charge in the MoH. However these changes took place not only at the higher political level, but also at the technical level, on the government side. And the on donors' side, there were also changes of staff at different levels (leadership and technical) due to their rotation policies. These changes highlight the volatility of the institutional aid environment where changes in persons (with their different personalities and motives) may impinge on the goals and strategies adopted.

## Discussion

### Interpreting motives through the lens of agency theory

**Figure 1:**
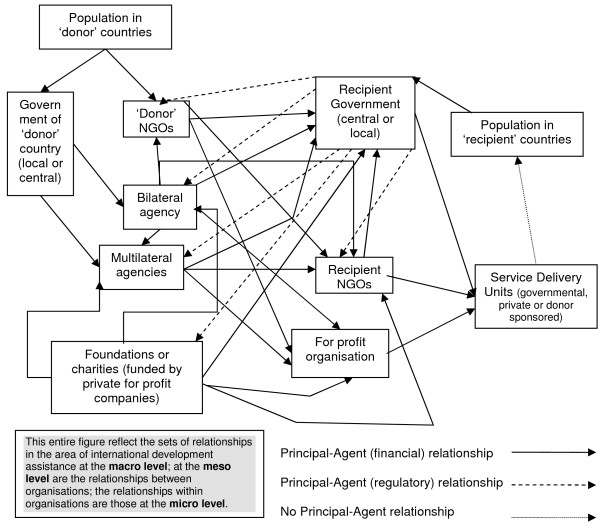
**Sets of principal-agent relationships in international development assistance**. Source: [47].

In trying to understand relationships in the aid environment, agency theory seems to provide some insights into the behaviour and motives of the actors involved. The international development assistance context contains various sets of principal-agent relationships between and within organizations [50], through multiple layers of delegation [29]. Figure 1 shows the main sets of principal-agent relationships in this environment. These are described as follows:

"*In a standard official bilateral aid setting, the chain of principal agent relationships starts with taxpayers as principals, who wish to transfer part of their income to recipients in other countries. They delegate the implementation of this transfer programme to their representatives (parliamentarians, politicians) who become their agents. These agents, in turn, become the political principals to an aid agency in charge of implementation of aid programmes. Within the aid agency, a hierarchical command chain creates a series of principal-agent relationships. When actual implementation is subcontracted to a private consultant or aid services supplier company, the task manager in the aid agency becomes a principal to the contractor; the latter becomes an agent to the task manager. Depending on the contract, the contractor may also be an agent to the recipient agency or counterpart administrator in the beneficiary country. The contractor may end up being an agent to two principals-a typical joint delegation situation. The recipient agent, in turn, is an agent to political principals and the beneficiary population in the recipient country*." [[29]: p. 18]

There are a number of variations to the model described above. A bilateral aid agency (like the UK Department for International Development) providing aid directly to a recipient government acts or tries to act as an agent on behalf of its government, and may in some respects act or try to act as the principal towards the recipient government. Another option is for governments (principals) to provide aid via multilateral development agencies (such as the World Bank) (agents).

In this context the system of accountability (or the principal's ability to ensure the advance of its objectives) is weakened as principals have to rely on the various layers of international bureaucracy and chains of principal-agent relationships to monitor and adjust penalties and rewards to performance.

Incentives serve to align conflicting objective functions. The GHIs appear to have created incentive mechanisms that have realigned Ugandan government objectives. This suggests GHIs operating in the role of principal vis-à-vis the Ugandan government, even if at the same time acting as 'agents' of their own 'principals' (for example their donor constituency-the US government, or President's office in the case of PEPFAR, a mix of bilateral and multilateral funding agencies in the case of the Global Fund).

The (vertical) project approach, as preferred by the GHIs examined by this study, has been identified with short term time horizons, the attribution of results directly to investments and greater control over financial management [51]. While fiduciary requirements are usually part of a SWAp or GBS agreement these may be less effective than the detailed scrutiny that can be exercised over projects. Precisely because a project is accountable for a single or limited number of outcomes and can ignore broader development objectives, it can operate in an 'insulated' environment and 'buy out' local constraints and uncertainties regarding disbursement bottlenecks and onerous bureaucratic controls [52]. All these arguably accord with the demands of political processes in donor countries: democratic government mandates usually of between 4 and 5 years [52], and the need to present success (and avoid scandal) to sustain popular consent to aid. SWAp shifts control from donors to recipient governments.

As agents, GHIs' incentives require them to invest and report on programs in a manner consistent with donor government objective functions and as principals, they pass on those incentives. The pursuit of short term and visible achievements may thus lead them to prioritize "*visible and uncontroversial forms of assistance with short-run payoffs.....rather than those with longer-run returns, like institutional reform*" [[53], p.20] which would be more compatible with the alignment agenda expressed by the Paris Declaration.

In themselves these insights fail to explain the conflict among different aid mechanisms and with the Paris Declaration. The mix of bilateral and multilateral agencies contributing to SWAp, general budgetary support and the Global Fund are significantly overlapping and these together with the US Government that is a signatory of PEPFAR are all signatories to the Paris Declaration. Insight into the motivations of the super-national level actors of those agencies is outside the scope of this research, but other studies have suggested that similar processes to those suggested at national level by which individuals and groups within these agencies with differentiated objective functions have variable degrees of authority over different operations of the agencies apply: "*as a whole, these principals, may have for a collective objective the maximization of the same social welfare function as that of a single benevolent regulator. However, each, single principal has only a limited mandate to fulfil*" [54].

Agency theory would also suggest that differences in the degree to which ultimate principals-tax payers in donor countries and different constituencies among them exert oversight over different operations will also be reflected in the incentives created. The influence achieved by some HIV/AIDS advocacy groups over a number of donor governments' operations can provide explanation both of the creation of the Global Fund and the particular political exigencies that govern its contract with its funders. The specific rather than general political origins of PEPFAR are similarly more likely to explain the incentive mechanisms it has created. In addition, to advocacy groups, both PEPFAR and the Global Fund have seen private sector and other civil society organisations permeate their values and structures thus also justifying the incentive regime created by these agencies. While the findings of this paper argue that the incentives in place led to parallel structures and fragmentation of actions, there is scope to interpret these incentives as catalysers of diversity in the form of public-private partnerships for example.

Agency theory also suggests that incentives have lower power where multiple principals compete for the effort of an agent [55]. The simplified institutional external aid scenario depicted in Figure 1 highlights the complexity of the aid system. There are not only multiple actors such as the UK and US governments but multiple mechanisms operated by the same governments-all competing for the weak institutional capacities of the Ugandan health system.

These two governments alone directly operate aid programs through DFID and USAID, contribute to the Global Fund, other GHIs such as GAVI, and multilateral agencies such as the World Bank and the World Health Organization, while the US government operates PEPFAR through separate mechanisms. The multiplicity of mechanisms add links in the chain of accountability from the ultimate principles in donor and recipient countries (tax payers and intended beneficiaries of aid) and obfuscate accountability through shared and therefore diluted responsibility for the effects of any given mechanism.

## Conclusions

This study seeks to contribute to the international debate on GHIs. While others found similar results in relation to the behaviour of GHIs during the earlier years of their operation [15,23], this paper provides greater explanation and new insights in relation to the motives of GHIs, other actors in aid transactions, and constituencies within the government of Uganda and how those motives are reflected in the incentives created by aid mechanisms and reaction to those incentives. Explanation and insight are the strengths of in-depth case study that have here been supplemented by elements of a historical perspective and the application of agency theory.

Agency theory helped to understand the impact of GHIs on the overall health aid scenario in Uganda, rather than specific elements of it by highlighting how they changed the way that incentives realigned the objective functions of principals and agents throughout the agency chain of development aid. But GHIs were not the only explanatory factor in the realignment that took place in Uganda. The acceptance of less integrated and nationally led aid required also a realignment of power between constituencies within the Ugandan government. The two sets of forces acted in tandem to change the aid contract.

This insight does not in itself provide solutions. A key problem in this environment is how to provide incentives that are sufficient in minimizing conflicts between the parties and lead to behaviours that result in a maximization of aid effectiveness. Underlying this problem are critical environmental complexities:

- Numerous layers of principal-agent relationships and actors and mechanisms simultaneously acting as principals and agents, weakening accountability links.

- Multiple, shifting and conflicting objectives that lead to difficulties in predicting how incentives will align and how they will be reacted to;

- Weak institutional and governance environments on both sides of the aid contract that result in poor alignment of activity within relevant organizations as well as between them.

Further research into the above areas could provide some insights at least at country level on some of the possible ways of mitigating key agency problems encountered in aid relationships. These could include for instance an analysis of how different incentives could work to align or re-align motives. Additional research focusing on institutional and governance environments (e.g. leadership and ownership) could contribute to understanding reforms and eventually bring in new ideas on how to strengthen these.

Since fieldwork, further shifts have occurred within GHIs, and at least some of them are supportive of better co-ordination and recipient government leadership and learning from the kinds of problems that have been documented here [7,9]. For instance, the Global Fund increased its use of country level procurement systems from 33% in 2005 to 56% in 2007; and monitoring and evaluation systems from 73% in 2005 to 82% in 2007 [56]. If the new government in the US proves to be more sympathetic to the harmonization and alignment agenda, PEPFAR may also evolve further in these directions.

Yet much needs to be done in order to render greater accountability of GHIs to their ultimate principals. To this end, Riddell [57] proposes a new international aid office to oversee all aid disbursements. Such a body would enforce sanctions in case of donors' misbehaviour. However, it is not clear from where the authority to sanction could emanate. Current global health governance is characterized by a large number of powerful actors with different interests, values, and mandates [58] suggesting that the creation of a regulatory body for aid is likely to be a politically elusive goal. In its absence, current proposals such as the International Health Partnership + and the peer review system established by the DAC/OECD could serve to provide more independent feedback to ultimate principals than is currently available and thus increase pressure on donors to honour commitments, if the political impetus to establish and empower such mechanisms can be mobilized.

## Competing interests

The authors declare that they have no competing interests.

## Authors' contributions

VOC and BM made substantial contributions to the conception and design of the study, as well as the acquisition, analysis and interpretation of data. VOC took the lead in writing the first draft of the manuscript and BM critically reviewed it. VOC and BM have both given final approval of the version to be published.

## Endnotes

^1^This amount includes four projects with duration of three years each.

^2^Total donor support to the general budget was US$275.1 million in 2003/4 [49]. In comparison donor support to the health sector was US$136.5 million in 2004/5 and the Global Fund average annual contribution was US$53.6 million - calculated on the basis of US$160.6 million allocated to four projects signed between 2003 and 2004 with duration of three years each, as reported earlier.
